# Parkinson-causing mutations in LRRK2 impair the physiological tetramerization of endogenous α-synuclein in human neurons

**DOI:** 10.1038/s41531-022-00380-1

**Published:** 2022-09-16

**Authors:** Luis Fonseca-Ornelas, Jonathan M. S. Stricker, Stephanie Soriano-Cruz, Beatrice Weykopf, Ulf Dettmer, Christina R. Muratore, Clemens R. Scherzer, Dennis J. Selkoe

**Affiliations:** Ann Romney Center for Neurologic Diseases, Department of Neurology, Brigham and Women’s Hospital and Harvard Medical School, Boston, MA 02115 USA

**Keywords:** Proteins, Cell biology

## Abstract

α-Synuclein (αSyn) aggregation in Lewy bodies and neurites defines both familial and ‘sporadic’ Parkinson’s disease. We previously identified α-helically folded αSyn tetramers, in addition to the long-known unfolded monomers, in normal cells. PD-causing αSyn mutations decrease the tetramer:monomer (T:M) ratio, associated with αSyn hyperphosphorylation and cytotoxicity in neurons and a motor syndrome of tremor and gait deficits in transgenic mice that responds in part to L-DOPA. Here, we asked whether LRRK2 mutations, the most common genetic cause of cases previously considered sporadic PD, also alter tetramer homeostasis. Patient neurons carrying G2019S, the most prevalent LRRK2 mutation, or R1441C each had decreased T:M ratios and pSer129 hyperphosphorylation of their endogenous αSyn along with increased phosphorylation of Rab10, a widely reported substrate of LRRK2 kinase activity. Two LRRK2 kinase inhibitors normalized the T:M ratio and the hyperphosphorylation in the G2019S and R1441C patient neurons. An inhibitor of stearoyl-CoA desaturase, the rate-limiting enzyme for monounsaturated fatty acid synthesis, also restored the αSyn T:M ratio and reversed pSer129 hyperphosphorylation in both mutants. Coupled with the recent discovery that PD-causing mutations of glucocerebrosidase in Gaucher’s neurons also decrease T:M ratios, our findings indicate that three dominant genetic forms of PD involve life-long destabilization of αSyn physiological tetramers as a common pathogenic mechanism that can occur upstream of progressive neuronal synucleinopathy. Based on αSyn’s finely-tuned interaction with certain vesicles, we hypothesize that the fatty acid composition and fluidity of membranes regulate αSyn’s correct binding to highly curved membranes and subsequent assembly into metastable tetramers.

## Introduction

Parkinson’s disease (PD) is the second most common neurodegenerative disorder. According to recent projections, it will affect more than 1 million people in the US alone by 2030^1^. PD progressively alters brain function to induce resting tremor, bradykinesia, postural instability, and rigidity, which arise due to an initially selective loss of nigrostriatal dopaminergic neurons followed by more widespread neurodegeneration, including in the cerebral cortex^2^. Though historically considered an idiopathic (‘sporadic’) disorder, at least 5–10% of PD cases are caused by mutations in certain genes, of which α-synuclein (*SNCA*) and leucine-rich repeat kinase 2 (*LRRK2*) are two of the most prominent hits in genome-wide association studies (GWAS)^3^. Hyperphosphorylated, aberrantly-folded, and aggregated forms of αSyn – together with altered organelles and lipid membranes – comprise Lewy bodies, the diagnostic neuronal inclusions of both sporadic and familial PD^4,5^.

LRRK2 is a large multi-domain protein with both GTPase and kinase catalytic activities, and a WD40 domain that likely mediates protein–protein, protein–membrane, and protein–cytoskeleton interactions^6–8^. Highly penetrant PD-causing mutations of LRRK2 mostly fall into the two catalytically active regions of the protein and either decrease its GTPase activity, such as R1441C^9^, or increase its kinase activity^10,11^, like the most common mutation, G2019S^12^. Whereas LRRK2 cases present with typical clinical features of PD, at the histopathological level, the G2019S mutation is associated with a ~79% frequency of Lewy body formation, more prominent than the R1441C mutation, where Lewy bodies form in ~43% of carriers^13^. However, these mean percentages are based on only a small number of autopsied patients to date, and the prevalence of Lewy bodies and other αSyn-positive lesions in the LRRK2 form of PD remains to be defined.

Although the precise function of αSyn is still debated, it has become increasingly accepted that at least part of its function in neurons involves the regulation of vesicle trafficking, including during synaptic transmission, for example, by controlling the dilation of the exocytotic fusion pore^14^ and chaperoning the assembly of SNARE complexes^15,16^. αSyn has long been considered a ‘natively unfolded’ protein in aqueous solution^17^ and in cells^18^, despite the widely-replicated finding that unfolded recombinant αSyn readily adopts an α-helical conformation upon binding to the outside of certain highly curved lipid vesicles in vitro^19^. In 2011, our laboratory reported four lines of evidence that physiological αSyn in neurons and erythrocytes can also exist in part as α-helically folded tetramers that are more resistant to pathological aggregation than the unfolded monomer^20^. Evidence of cellular tetramers with a size and conformation distinct from the monomer was provided by (1) native gel electrophoresis; (2) intact-cell crosslinking; (3) STEM (scanning transmission electron microscopy); and (4) SE-AUC (sedimentation equilibrium analytical ultracentrifugation), which predicted a mass of tetramers purified from fresh human erythrocytes of 57,753 Da (4 monomers = 57,760)^20^. Because these biophysical methods are not widely accessible, we further developed our crosslinking protocol using the cell-penetrant, lysine-directed crosslinker disuccinimidyl glutarate (DSG: required atomic spacing between lysines: 7.7 A) to detect the tetramers (~60 kDa) by SDS-PAGE/Western blotting^21^. This method usually reveals minor αSyn-immunoreactive bands at ~80 and ~100 kDa that appear to be conformers of the ~60 kDa tetramer^21^.

As regards disease relevance, we showed that all fPD-causing missense mutations in αSyn lower the stability of the tetramers, resulting in decreased tetramer:monomer (T:M) ratios in intact neural cells^22^. Some αSyn mutations lowered the T:M ratio and led to the formation of round, vesicle-rich, αSyn-immunoreactive inclusions associated with neurotoxicity^22^. Moreover, we showed that transgenic (tg) expression in mice of the E46K mutation that causes PD and dementia with Lewy bodies (DLB), and especially expression of an engineered mutant with two additional E→K substitutions in the adjacent KTKEGV repeat motifs (E35K + E46K + E61K), can produce a striking PD-like motor phenotype with resting tremor and limb and gait deficits responding in part to L-DOPA^23^. These ‘tetramer-abrogating’ tg mice have nigral and cortical αSyn inclusions, increased αSyn pSer129 phosphorylation, decreased dopamine levels, disrupted synaptic ultrastructure, and lysosomal changes resembling those of human PD^16,23,24^.

Other groups have also described physiological multimeric forms of αSyn that participate in synaptic vesicle activity during SNARE-complex formation^25^ and exocytotic events^26,27^. Accordingly, we hypothesize that αSyn exists in an intracellular equilibrium between monomeric and tetrameric/multimeric forms in healthy cells and can be shifted toward more aggregation-prone monomers in PD.

Like PD-associated mutations of αSyn^22^, loss-of-function mutations in glucocerebrosidase (*GBA*), which cause Gaucher’s disease but also predispose carriers to PD^28^, have recently been shown to partially shift the T:M ratio toward monomers^29^. iPSC-derived human neurons from GBA mutation carriers showed a 30-40% decrease in T:M ratio and a concomitant accumulation of monomers; here, αSyn has the wild-type (WT) sequence and is endogenously expressed^29^. Together, accumulating evidence suggests that αSyn exists in an equilibrium between unfolded monomers and α-helically folded tetramers in cells under physiological conditions. Skewing this ratio toward a more monomeric state – either by mutations in αSyn itself or by alterations in a PD-causing pathway upstream of it (*GBA* dysfunction) – results in a pathogenic state that promotes hyperphosphorylation and aggregation of αSyn and eventual neurotoxicity.

Given that LRRK2 dysfunction caused by G2019S and other mutations correlates with extensive Lewy body formation in the brains of many carriers autopsied to date^13^, we hypothesized that LRRK2 dysfunction could alter the homeostasis of αSyn, specifically its tetramer:monomer equilibrium. To answer this previously unaddressed question about PD pathogenesis, we quantified the multimerization of αSyn in induced pluripotent stem cell (iPSC)-derived, neurogenin-induced human neurons (called iNs) from PD patients with the most prevalent LRRK2 mutations, G2019S (referred to as LRRK2 line (L) 1 and L2 hereafter) and R1441C (referred to as lines L3 and L4). We found that both LRRK2 mutations significantly reduced the T:M ratio of endogenous, WT αSyn in the cytoplasm of the intact neurons and thereby promoted the accumulation and pSer129 phosphorylation of αSyn monomers. The findings critically expand the concept that the normal state of αSyn requires its ability to continuously form metastable tetrameric assemblies and that factors other than its own sequence can adversely affect this equilibrium. Mechanistically, we show that two distinct LRRK2 kinase inhibitors^30,31^ can reverse the αSyn alterations in the G2019S and the R1441C neurons. Importantly, a stearoyl CoA desaturase (SCD) inhibitor, which has been shown to improve αSyn dyshomeostasis in cultured neurons^32,33^ and mice^34^ expressing fPD-mutant αSyn, rescued the decreased T:M ratio for both LRRK2 mutations, supporting a common pharmacological treatment for tetramer-abrogating forms of PD.

## Results

### The LRRK2 G2019S mutation destabilizes the physiological tetramer:monomer equilibrium of αSyn and increases phosphorylation at pSer129, a marker of PD

**Fig. 1:**
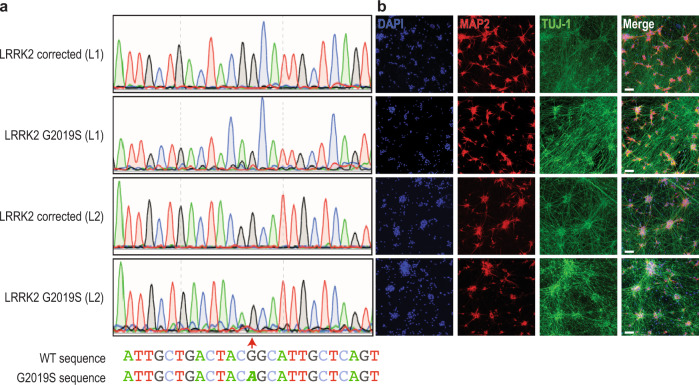
Characterization of two G2019S-mutant LRRK2 patient neuronal lines and their isogenically corrected versions. **a** Genomic DNA sequencing showing the presence of heterozygous G2019S mutation (guanine to adenosine transition, red dashed arrow) in two G2019S patient-derived iNs (L1, L2) but not in their isogenically corrected versions. **b** Representative confocal images of neurogenin-induced iPSCs (iNs) at DIV 14 showing DAPI-stained nuclei in blue and the neuronal markers MAP2 and TUJ-1 in red and green, respectively. White bars: 20 μm.

**Fig. 2:**
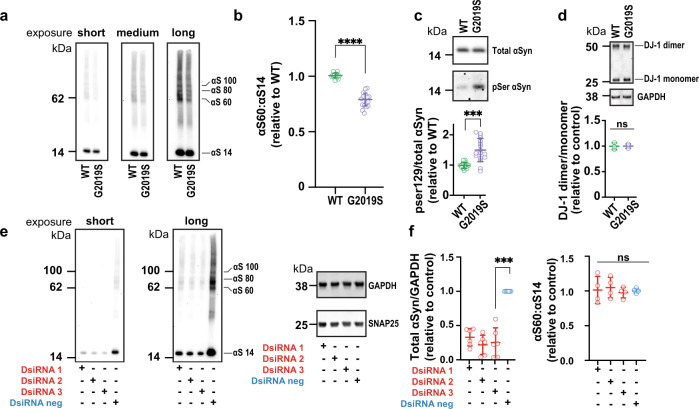
LRRK2 PD-causing mutations decrease αSyn tetramer:monomer ratios and increase monomer phosphorylation in iPSC-derived patient neurons (iNs). LRRK2 G2019S mutation in each of two different patient lines decreases the endogenous αSyn tetramer (αS60, numerator) to monomer (αS14, denominator) ratio. **a** Representative WB of total lysates prepared after intact-cell crosslinking with 1 mM DSG. Each G2019S patient line has an isogenically corrected (WT) line. **b** Quantification (*N* = 2 patient lines; *n* ≥ 5 experiments) of the αS60:αS14 (T:M) ratio in the mutant vs. WT lines. **c** While total αSyn levels are unchanged by the G2019S mutation, it significantly enhances pSer129 phosphorylation (*N* = 2 patient lines; *n* ≥ 3). **d** DJ-1 WB of the same lysates as in A shows uniform crosslinking of the endogenous DJ-1 dimers, excluding differential crosslinking efficiency as the reason for the abnormal αSyn T:M ratio. **e** Three distinct DsiRNAs to αSyn show that the multimeric bands (αS60, αS80, αS100) are specific αSyn species and do not alter general protein expression (shown are GAPDH and SNAP25). **f** While the total αSyn levels are significantly reduced by all 3 DsiRNAs, the αSyn T:M ratio is not altered. (*N* ≥ 8 independent experiments; each dot is the average quantification of an independent six-well plate experiment; one-way ANOVA with Tukey’s post hoc test; error bars: SEM; ****p < 0.0001; ***p < 0.001; ns not significant).

To assess whether PD-causing LRRK2 dysfunction causes downstream effects on the normal multimerization of αSyn, we obtained iPSCs derived from two unrelated PD patients harboring the G2019S mutation from the Stem Cell Engineering facility at CMCB (Technische Universität Dresden), referred to here as G2019S L1 and G2019S L2^35^. We also obtained from them the gene-corrected iPSC lines corresponding to each patient line, referred to here as Corr. L1 and Corr. L2. DNA sequencing confirmed the presence of the G2019S mutation and its correction, respectively (Fig. 1a), and the cells had normal karyotypes (Supplemental Fig. 1). We established that the four LRRK2 lines responded well to the neuronal differentiation protocol^36^ we have used previously^37^: after 21 days of neurogenin 2 induction, the neuronal markers MAP2 and TUJ-1 displayed similar intensity and distribution across the four lines (Fig. 1b). In previous neuronal differentiation protocols, these same patient lines have been shown to exhibit increased phosphorylation of extracellular signal-regulated kinase (ERK) that reverted to normal levels with the correction of the G2019S mutation, as expected^35^. In order to trap and visualize the cell-lysis-sensitive intracellular tetrameric assemblies of αSyn^20,21^, we employed a well-established, intact-cell crosslinking method by applying a 1 mM solution of the cell-penetrant crosslinker DSG^21^. The iNs harboring the G2019S LRRK2 mutation consistently showed a decreased tetramer (αS 60) to monomer (αS 14) ratio of their endogenous αSyn compared to the isogenically corrected control neurons (Fig. 2a-left panel; quantified in 2B; see also Supplemental Fig. 3 showing western blots and quantification for both G2019S lines and their corrected control lines run side-by-side).

It is widely reported that pathogenic αSyn accumulation in PD correlates with increased phosphorylation of the serine residue at position 129^38^. Up to 90% of the αSyn within Lewy bodies in PD brains is hyperphosphorylated there^39,40^. We asked whether LRRK2 mutations that shift the T:M ratio toward more potentially self-aggregating monomers would also increase αSyn Ser129 phosphorylation. Analyses of the iN lines of the two patients with the G2019S LRRRK2 mutation revealed significant increases in the phosphorylation level of αSyn at Ser129 vs. their isogenic controls, while there was no difference in total αSyn levels (Fig. 2c).

To rule out differences in crosslinking efficiency as drivers of the reduced T:M ratio of αSyn, we probed for DJ-1 dimerization in the same samples (Fig. 2d). In accord with our previous results^21^ we found no differences in the dimer to monomer ratio of DJ-1. We confirmed the αSyn identity of the crosslinked multimeric bands by employing three different Dicer-Substrate siRNAs (DsiRNAs) targeting αSyn that lowered its expression between ~75% and 90% (Fig. 2e, quantified in f). The bands we quantify as tetrameric and monomeric αSyn were all proportionately decreased by the DsiRNAs, showing that these higher bands are indeed αSyn multimers trapped by the intact-cell DSG crosslinking. Notably, the αSyn T:M ratio was not significantly altered by lowering the αSyn level (Fig. 2f), as we reported previously^22^.

In the LRRK2 G2019S mutant lines L1 and L2, immunoblotting revealed no significant differences in the expression levels of the neuronal markers SNAP25 and PSD95 vs. the isogenic control lines (Supplemental Fig. 2). However, in agreement with an earlier report^41^, the LRRK2 mutant lines had significantly reduced expression of Rab11 (Supplemental Fig. 2), a key protein in vesicle trafficking, specifically in endocytic recycling^42^.

### The altered αSyn T:M equilibrium and pSer129 phosphorylation are restored by inhibiting the mutant LRRK2 G2019S kinase activity

**Fig. 3:**
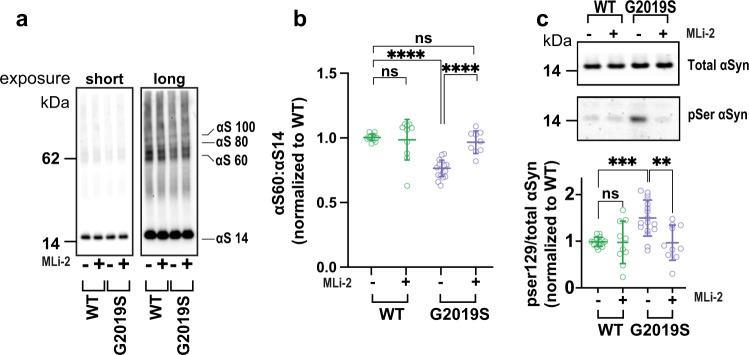
Decreased tetramer:monomer ratio and increased phosphorylation of αSyn are reverted by LRRK2 kinase inhibition. **a** Representative WBs of total cell lysates after intact-cell crosslinking following conditioning with (+) or without (−) MLi-2 inhibitor (0.01 μM). **b** Quantification shows that LRRK2 kinase inhibition restores the lowered αSyn T:M ratio caused by the G2019S mutation. **c** Quantification of phosphorylated and total αSyn in mutant vs. corrected neurons with (+) and without (−) MLi-2 treatment. Inhibiting LRRK2 kinase restores normal αSyn phosphorylation (For both B and C, *N* ≥ 4 independent experiments; each dot is the average quantification of an independent six-well plate experiment; one-way ANOVA with Tukey’s post hoc test; error bars = SEM; **p < 0.01, ****p < 0.0001, ns not significant).

To assess whether mechanistically the decrease in αSyn T:M ratio was attributable to the enhanced kinase activity widely reported to be conferred by the G2019S mutation, we treated the neurons with two highly specific LRRK2 kinase inhibitors, PF-06447475 (henceforth referred to as PF)^30^ or MLi-1^31^. In accord with previous studies^43^, treating our LRRK2 iNs with a 0.5 μM concentration of the PF compound or 0.01 μM of the MLi-2 compound during 8–15 days of neuronal induction yielded no toxic effects on the cells. We found that the reduced αSyn T:M ratio caused by the LRRK2 G2019S mutation was fully prevented by treatment with MLi-2 (Fig. 3a, b) or PF (Supplemental Fig. 3). To assess whether the increased levels of phosphorylated αSyn in the LRRK2 mutant neurons were associated with their lowered tetramerization propensity, we quantified the pSer129 levels of the MLi-2-treated cells. Upon 8 days of treatment, MLi-2 significantly reduced the overall amount of pSer129 phosphorylated αSyn in the G2019S mutant lines without affecting total αSyn levels (Fig. 3c). The LRRK2 kinase inhibitor had no effect on total or pSer129 αSyn in the absence of the PD-causing mutation (Fig. 3c).

To support this line of evidence, we examined the phosphorylation levels of a widely accepted substrate of LRRK2 kinase activity, Rab10^44,45^. In agreement with some previous studies^10,44–48^, we observed enhanced phosphorylation of Rab10 at position Thr-73 for both G2019S and R1441C mutants of LRRK2 (Supplemental Fig. 4). The phosphorylation was significantly diminished in both mutants and isogenically corrected cells (Supplemental Fig. 4) treated with MLi-2, confirming its ability to prevent LRRK2-mediated phosphorylation of endogenous Rab10 in our human neurons^49,50^.

### Fatty acid metabolism has a regulatory role in the LRRK2-mediated alteration of αSyn tetramerization and phosphorylation

**Fig. 4:**
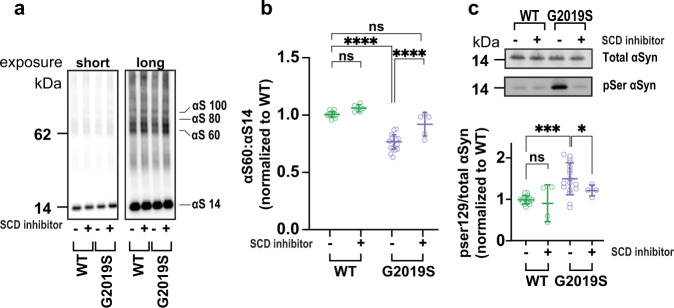
Reduced tetramer:monomer ratio and increased pSer129 phosphorylation of αSyn in LRRK2 G2019S neurons are rescued by SCD inhibition. **a** Representative WBs of total cell lysates after intact-cell crosslinking following conditioning with (+) or without (−) 5b compound (0.5 μM). **b** Quantification shows that inhibiting SCD increases the lowered αSyn T:M ratio generated by the G2019S mutation. **c** Quantifying phosphorylated and total αSyn in G2019S neurons with (+) and without (−) 5b treatment. Inhibiting SCD restores normal levels of αSyn phosphorylation (For both **b** and **c**, *N* ≥ 4 independent experiments; each dot is the average quantification of an independent six-well plate experiment; one-way ANOVA with Tukey’s post hoc test; error bars = SEM; *p < 0.05, ***p < 0.001, ****p < 0.0001, ns not significant).

Altered lipid and fatty acid homeostasis has emerged as a mechanistic feature and pharmacological target in synucleinopathies^4,32,51–53^. We previously showed that decreased T:M ratios caused by fPD αSyn mutations in cultured neurons and transgenic mice could be prevented by genetically or pharmacologically downregulating stearoyl-CoA desaturase (SCD), the rate-limiting enzyme for converting saturated to monounsaturated fatty acids^32,33,54^. Accordingly, we treated the LRRK2 G2019S mutant and corrected iNs with a well-studied commercial SCD inhibitor referred to as 5b^34,55^. Conditioning the G2019S LRRK2 mutant neurons with 0.5 μM 5b for 8 days during iN differentiation (DIV 5-14) restored their physiological T:M ratio while having no effect on the normal T:M ratio of the isogenically corrected wild-type neurons (Fig. 4a; quantified in 4B; see also Supplemental Fig. 3). Like our MLi-2 treatment, SCD inhibition with 5b also significantly reduced the phosphorylation levels of αSyn at Ser129 (Fig. 4c). Treating the neurons for only ~12 h with either MLi-2 or 5b failed to restore the T:M ratio of αSyn (Supplemental Fig. 5; see Discussion section).

### The R1441C PD-causing LRRK2 mutation also decreases the αSyn T:M ratio and increases pSer129 phosphorylation

**Fig. 5:**
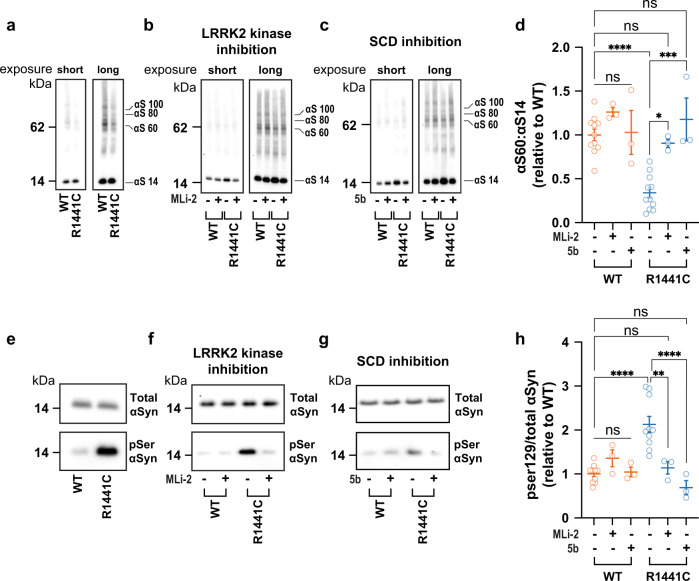
The R1441C LRRK2 mutation also decreases the physiological αSyn T:M ratio. **a** Representative WBs of patient lines bearing the LRRK2 R1441C mutation show significantly decreased αSyn T:M ratios. Treatment with either a LRRK2 kinase inhibitor (MLi2) (**b**) or an SCD inhibitor (5b) (**c**) restored the physiologic T:M ratio WT levels **d**. **e** R1441C also increases pSer129 phosphorylation of αSyn. **f** Inhibiting LRRK2 kinase or **g** inhibiting SCD reduced pSer129 levels. **h** Quantifying the ratio of pSer129 to total αSyn (for both **d** and **h**, *N* ≥ 3 independent experiments; each dot is an independent replicate sample; one-way ANOVA with Tukey’s post hoc test; error bars = SEM; *p < 0.05, **p < 0.01, ***p < 0.001, ****p < 0.0001).

We next asked whether the R1441C mutation, which is in the GTPase domain of LRRK2^9^, has similar effects on αSyn homeostasis as the G2019S mutation has. After 21 days of differentiation, the LRRK2 R1441C mutation in two independent patient-derived neuronal lines (L3 and L4) also reduced the T:M ratio of αSyn consistently and significantly (Fig. 5a, d).

Treatment with each of the two LRRK2 kinase inhibitors restored the normal T:M ratio of αSyn in the R144C lines (MLi-2: Fig. 5b, d; PF: not shown), highlighting the relevance of LRRK2 kinase activity on the homeostasis of αSyn. Inhibition of SCD with compound 5b also rescued the αSyn T:M and pSer129 abnormalities (Fig. 5c, d, g, h). None of these three compounds had significant effects on the isogenic WT neurons (Fig. 5d).

**Fig. 6:**
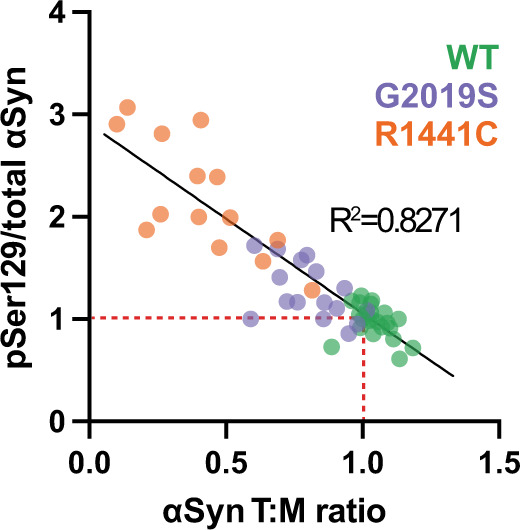
Significant (*p* < 0.0001) negative correlation between αSyn T:M ratio and pSer129 phosphorylation. Each dot represents a neuronal sample for which both the pSer129 level and the T:M ratio of αSyn was quantified (without any inhibitor treatment). WT controls (corrected lines also without inhibitor treatment) were normalized to 1 for both ratios (red dotted lines).

Finally, we analyzed the results from all of our iN experiments in which both the pSer129 phosphorylation level and the T:M ratio had been measured on the same cell sample. We observed a significant negative correlation between the relative levels of the αSyn T:M ratio and the relative levels of pSer129 phosphorylation across all of these experiments (Fig. 6). The lower (more abnormal) the T:M ratio in the human neurons was, the higher (more abnormal) the phosphorylation state of αSyn was.

### The LRRK2 G2019S mutation alters αSyn homeostasis in midbrain dopaminergic human neurons

**Fig. 7:**
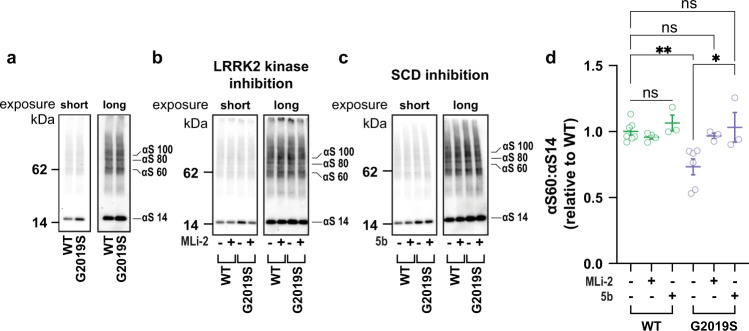
The G2019S LRRK2 mutation decreases the physiological αSyn T:M ratio in midbrain dopaminergic neurons. Patient-derived iPSCs carrying the G2019S mutation in LRRK2 and its isogenically corrected WT line were differentiated into midbrain DA neurons. **a** The G2019S mutation reduced the αSyn T:M ratio. Treatment with either a LRRK2 kinase inhibitor (**b**) or an SCD inhibitor (**c**) restored the physiological T:M ratio WT levels (**d**). (*N* ≥ 3 independent experiments; each dot is the mean of an independent replicate sample; one-way ANOVA with Tukey’s post hoc test; error bars = SEM; *p < 0.05, **p < 0.01, ns not significant).

To validate our results in a different cell type highly relevant to PD, we differentiated the same iPSC patient lines that carry the G2019S mutation to generate midbrain dopaminergic (mDA) neuronal cultures using a floor plate-based protocol^56,57^. While mDA neurons express LRRK2^58–60^, they do so at a significantly lower level than that of cortical glutamatergic neurons (iNs) (Supplemental Fig. 6). By using immunofluorescence, we found that cells in our culture that are positive for tyrosine hydroxylase also express LRRK2 (Supplemental Fig. 7). Although the effect was slightly more modest in mDAs than in iNs, we found that the G2019S mutation significantly decreased the αSyn T:M ratio (Fig. 7a, d). Also in agreement with the earlier results in iNs, treatment with either a LRRK2 kinase inhibitor (MLi-2) or an SCD inhibitor (5b) fully restored the physiological T:M ratio of αSyn in the LRRK2-mutant dopaminergic neurons (Fig. 7b–d).

## Discussion

Misfolded, aggregated forms of αSyn are the primary proteinaceous component of Lewy bodies, and missense mutations and duplication/triplication of the *SNCA* gene cause aggressive, early-onset forms of familial PD, strongly suggesting that αSyn plays a central role in the pathogenesis of PD and related synucleinopathies such as Lewy body dementia. We have proposed that physiological αSyn occurs in part as α-helical tetramers^20,21^ and that partial shifts of tetramers to more aggregation-prone monomers are associated with neurotoxicity^22,61^, and in mice, with striking PD-like, L-DOPA-responsive motor phenotypes^23^. The potential relevance of tetramer destabilization to PD was initially shown only for mutations in its sequence^22^. However, an advance for this hypothesis was the discovery that PD-associated mutations in GBA1 could likewise promote the destabilization of endogenous αSyn tetramers, indicating that genetic defects in proteins other than αSyn can affect its normal assembly state^29^. Here, we extend this evidence in an unexpected direction by showing that two pathogenic mutations of LRRK2, G2019S, and R1441C – among the more common genetic alterations to emerge from what was previously considered to be ‘sporadic’ PD – can consistently and significantly decrease the T:M ratio of WT αSyn at normal levels of endogenous expression in human neurons. Moreover, the shift toward relatively more monomers is accompanied by an increase in their phosphorylation at Ser129, a post-translational modification often observed in cultured cells and mouse models of PD, and importantly, in the Lewy bodies/neurites of PD patients^38,40^.

Besides its reported roles in the regulation of autophagy^62–64^, mitochondrial function, aging and cell death^35,65–68^, and neurite growth^63,69^, the G2019S mutation of LRRK2 has also been strongly associated with increased accumulation of αSyn^13,35,63,68^ and pathologic build-up of its Ser129-phosphorylated form^70^. In a PD mouse model, the G2019S mutation led to exacerbated αSyn pathology and dopaminergic neurodegeneration^71,72^. The R1441C mutation in LRRK2 also leads to parkinsonism that is clinically similar to sporadic PD^73^. Although it falls into the GTPase domain of the protein and is less frequent than its G2019S counterpart, the R1441C mutation is also associated with dysregulated phosphorylation of LRRK2 substrates^9,10,48^. In transgenic mice, R1441C LRRK2 leads to impairments in fine motor tasks, gait, olfaction, and synaptic transmission^74,75^.

Cell culture studies have shown that the upregulation of Rab11 can revert the vesicular trafficking defects generated by the widely documented hyperphosphorylation conferred by the G2019S LRRK2 mutant^41^. We found significantly reduced expression of Rab11 in our human G2019S and R1441C neurons (Supplemental Fig. 2), leading us to speculate that the elevated kinase activity of the LRRK2 mutants can generate an impaired vesicle trafficking environment that negatively affects the ability of αSyn monomers to interact correctly with the outer surface of small cytoplasmic vesicles (including synaptic vesicles) and efficiently become α-helical^19^ and tetrameric^20^. Inhibiting the phosphorylating activity of LRRK2 with two structurally distinct LRRK2 kinase inhibitors (MLi-2 and PF) largely prevented the deleterious effects of both LRRK2 mutations on αSyn (i.e., reduced T:M ratio and increased pSer129 phosphorylation). Collectively, our findings provide a clear link between PD-causing dysfunctions of the LRRK2 kinase and GTPase domains and altered αSyn tetramerization and phosphorylation.

Because treatment with the in vivo-validated SCD inhibitor 5b^34^ prevented the decreased T:M ratio of αSyn in both the G2019S and R1441C human neurons (as it does for fPD-mutant αSyn), we speculate that the mutant LRRK2-driven dyshomeostasis of αSyn may arise from altered fatty acyl composition of certain membranes that is important for proper transient αSyn-membrane binding and tetramer formation (discussed in ref. ^32^), as may also occur in the synucleinopathy caused by GBA1 loss-of-function mutations^29^. All pathogenic mutations in LRRK2 which have been tested increase Rab10 phosphorylation in vivo^76^. That endogenous levels of LRRK2 mutants in patient neurons promoted hyperphosphorylation of endogenous Rab10 (Supplemental Fig. 4), and that specific LRRK2 kinase inhibitors block that hyperphosphorylation and prevent the αSyn phenotypes support the idea that the alterations we observe in αSyn are a downstream effect of LRRK2 dysfunction. Together, these results indicate that the formation and stability of physiological tetramers of αSyn do not depend solely on the sequence of αSyn but also on the cytoplasmic environment to which WT αSyn is exposed. Increased αSyn membrane binding, as occurs with some (e.g., E46K) but not other (e.g., G51D) PD-causing mutations, chronic dyshomeostasis of lipid metabolism (as in GBA1 loss-of-function mutations), and membrane trafficking defects (as have been reported for the G2019S and R1441C LRRK2 mutations^41,77,78^) have now each been associated with a statistically significant shift of physiological tetramers toward αSyn monomers, which can then become hyperphosphorylated and potentially aggregated. Indeed, we show here that the T:M ratio of αSyn correlates quantitatively with its pSer129 phosphorylation: lower T:M ratios are associated with more monomer phosphorylation (Fig. 6). Because elevated pSer129 phosphorylation has been found in many studies to occur in PD brain (including in Lewy bodies/neurites) and PD mouse and neuronal culture models, lower T:M ratios appear to arise in conditions in which cellular homeostasis has been compromised – either by genetic changes in αSyn sequence or amount or by non-αSyn genetic abnormalities that predispose humans to PD. The fact that we observed the reduction of αSyn T:M ratio in both cortical and dopaminergic cell types carrying the same LRRK2 mutation underscores the consistent biochemical relationship between LRRK2 dysfunction and altered αSyn multimerization and phosphorylation. We note that we observed a higher phosphorylation of Rab10 in the R1441C than the G2019S LRRK2 mutant cell lines (Supplemental Fig. 4), in accord with previously reported results^44,45^. We believe that this enhanced effect may be related to the more pronounced reduction in the T:M ratio of αSyn in the R1441C than G2019S mutant cell line (Fig. 5).

Our findings support further screening for small molecules which reestablish the physiological T:M ratio^54^ as a rational disease-modifying strategy for PD and DLB. Indeed, our observations that well-characterized pharmacological inhibitors of LRRK2 kinase or of SCD, the rate-limiting enzyme in monounsaturated fatty acid biosynthesis, can each fully ameliorate the T:M shift and abnormal Ser129 phosphorylation of monomers in human neurons suggest that the recent entry of an SCD inhibitor into clinical trials in ‘sporadic’ PD^79^ may also be relevant to the LRRK2 form of the disease. Intensive studies of human neurons and mouse models expressing PD-causing LRRK2 mutations will now be needed to establish the molecular mechanism by which the mutants result in this αSyn dyshomeostasis.

At least four distinct biochemical methods (summarized in the Introduction) have demonstrated the existence of αSyn tetramers in normal cells and brain. Importantly, subsequent molecular modeling indicated that the tetramer is an energetically favored form of the protein^80,81^. The central conclusion of our study is that LRRK2 mutations can lower the physiological T:M equilibrium of endogenous, wild-type αSyn in human neurons bearing such PD-causing mutations. Thus, mutant LRRK2 joins two other dominant genetic forms of PD, mutant αSyn and mutant GBA, in sharing a biochemical mechanism that can occur upstream of progressive neuronal synucleinopathy in these disorders. Because of αSyn’s delicate and finely-tuned interaction with membrane lipids, we favor the hypothesis that membrane fluidity (e.g., MUFA and SFA levels) are crucial factors that help regulate αSyn’s correct binding to certain curved membranes and subsequent assembly into metastable tetramers. The mechanism by which the compounds used in our study restore the proper αSyn T:M ratio is likely to be indirect, given that compound treatment of just 12 h failed to revert the pathogenic phenotype (Supplemental Fig. 5) whereas 8 day treatment consistently did. Stabilizing the tetrameric state of αSyn, including via lipidomic strategies such as upregulating GBA^24^ or inhibiting SCD^79^, should be actively pursued for both familial and ‘sporadic’ forms of PD and related human synucleinopathies. Indeed, αSyn’s ability to bind transiently to vesicle membranes may represent a crucial aspect in its demonstrated necessity to populate diverse and dynamic structural arrangements. An approach seeking to stabilize tetrameric assemblies would be analogous to the clinically successful stabilization of the physiological transthyretin tetramer by the FDA-approved small molecule tafamidis for cardiac and neural transthyretin amyloidosis^82^.

## Methods

### Ethics

All studies on human cell lines were given ethical approval by the Institutional Review Board of the Mass General Brigham healthcare system (#2018P000009). No living participants were involved in this study, only iPSC cell lines that were received from outside central repositories, as indicated below. All participants who provided samples for the generation of iPSC lines used in this study had signed written informed consent to provide their cells.

### Induced neuron generation

iPSCs from the G2019S LRRK2 lines 1 and 2, with their respective isogenically corrected lines were obtained from the Stem Cell Engineering facility at CMCB (Technische Universität Dresden with all MTAs in place)^36^. The R1441C LRRK2 lines 3 and 4 were obtained from NINDS (all MTAs in place) and were isogenically corrected to WT LRRK2 using a CRISPR-Cas9 system (Synthego, Redwood City, CA). All iPSCs were used to prepare neurogenin 2 (Ngn2)-induced human neurons^36^. iPSCs were maintained in media containing DMEM/F12, Knockout Serum Replacement, penicillin/streptomycin/glutamine, MEM-NEAA, and 2-mercaptoethanol (all from Invitrogen, Carlsbad, CA) with addition of 10 μg/mL bFGF (Millipore, Billerica, MA) directly prior to media application. Neuronal differentiation was performed via a doxycycline-induced neurogenin 2 system. iPSCs were plated at a density of 95,000 cells/cm^2^ for viral infection. Lentiviruses were obtained from Alstem with “ultrapure titers” and used at the following concentrations: pTet-O-NGN2-puro: 0.1 μl/ 50,000 cells; Tet-O-FUW-eGFP: 0.05 μl/ 50,000 cells; Fudelta GW-rtTA: 0.11 μl/50,000 cells. To induce Neurogenin 2 expression, doxycycline is added on “iN day 1” at a concentration of 2 μg/ml. On iN day 2 puromycin is added at 10 mg/ml and is always maintained in the media thereafter. On iN day 4, cells were plated at ~75,000 cells/cm^2^ on matrigel (BD Biosciences, San Jose, CA)-coated Greiner 6, 24, or 96 well microclear plates and maintained in media consisting of Neurobasal medium (Gibco), Glutamax, 20% Dextrose, MEM NEAA with B27, with BDNF, CNTF, GDNF (PeprpTech, Rocky Hill, NJ) each at a concentration of 10 ng/ml. At designated time points, iNs were characterized using live-cell imaging to monitor neuritic complexity and used for immunocytochemistry (Fig. 1b) or Western blotting to assess the expression of neuronal markers (Supplemental Fig. 2). For experiments investigating the effects of the G2019S and R1441C mutations of LRRK2, iNs were used at iN day 21, a time point when they were fully mature.

### Midbrain dopaminergic differentiation

The G2019S L2 line and its respective isogenic control were differentiated according to a slightly modified version of ref. ^57^. Briefly, iPSCs were seeded at a density of 200,000 cells/cm^2^ and exposed to LDN19318 (Stemgent, Beltsville, MD), SB431542 (Stemcell Technologies, Cambridge, MA) high levels of SHH by combining ShhC25II (R&D systems, Minneapolis, MN) with Purmorphamine (EMD Millipore, Burlington, MA), FGF8 (Peprotech, Rocky Hill, NJ) and timed activation of the Wnt signaling pathway using the small molecule CHIR99021 (Miltenyi Biotech, Cambridge, MA). The Knockout Serum Replacement/ Knockout DMEM medium (Gibco) was gradually replaced with Neurobasal medium every other day starting d5. At d11 medium was changed to Neurobasal/B27/GlutaMAX containing CHIR99021. Cells were replated in a 1:1 ratio on Matrigel-coated dishes in Neurobasal/B27/GlutaMAX medium containing BDNF, GDNF, TGF-ßIII (all Pepro Tech, Rocky Hill, NJ), L-ascorbic-acid (MilliporeSigma, Burlington, MA), dibutyryl-cAMP (Enzo Life Sciences Inc, Farmingdale, NY) and supplemented with Y27632 (Stemcell Technologies, Cambridge, MA) on d13. Cells were plated between d20-d24 on Poly-D-lysine (MilliporeSigma, Burlington, MA) laminin (R&D systems, Minneapolis, MN) coated plates and exposed to 3 µM cytosine arabinoside (MilliporeSigma, Burlington, MA) for 2 days. Around d30, cells were dissociated using Accutase (Gibco) supplemented with Y27632 and plated at a density of 250,000 per well onto 24-well plates (Corning, Tewksbury, MA). After final plating, media was changed every 2-3 days.

### Small compound treatment. Neurogenin induced neurons

A 0.5 μM concentration of PF-06447475 or 300 nM concentration of MLi-2 inhibitors of LRRK2 kinase activity, or a 0.5 μM concentration of the SCD inhibitor 5b were added to a fresh media change on day 6 after neuronal induction. Subsequent media changes included the same concentration of the compounds and were performed every 2-3 days until cell harvesting. Dopaminergic neurons: At d50, neurons were treated with 0.01 µM of MLi-2 for LRRK2 kinase activity inhibition, 0.5 µM of the SCD inhibitor 5b or DMSO as vehicle control every 2–3 days until harvesting at d60.

### Cross-Linking

DSG (1 mM) was used to trap multimeric species of αSyn. For iNs, in-well cross-linking was performed in 6-well plates: cells were washed with 800 μL of HBSS. Then, 800 μL of 1 mM DSG in HBSS were added to each well, incubated at 37°C for 30 min, and quenched with Tris HCl at a final concentration of 20 mM for 15 min at room temperature.

### Western blot and immunocytochemical characterization of induced and dopaminergic neurons

Cells grown in six-well plates were lysed by the addition of 50 μl lysis buffer (0.1% SDS, 1% NP-40, 50 mM HEPES, pH 7.4, 2 mM EDTA, 100 mM NaCl, 5 mM Na_3_VO_4_, 40 μM p-nitrophenyl phosphate, and plus protease inhibitors) and incubated at 4 °C for 30 min. Lysates were centrifuged at 15,000×*g* and 4 °C for 30 min in a benchtop Eppendorf centrifuge. Supernatants were collected and protein content was determined using a BCA kit (Thermo Fisher Scientific, Waltham, MA). 20 μg of total protein (50–75 μg in the case of LRRK2 of dopaminergic neurons, as well as Rab10 and pRab10 blots from iNs) was loaded in each lane and electrophoresed on pre-cast 15 well 4–12% polyacrylamide Bis-Tris LDS gels (Invitrogen, Carlsbad, CA). Proteins were transferred onto 0.2 μm nitrocellulose, and blots were incubated overnight at 4°C with primary antibody. Membranes were washed three times for 5 min with PBST (or TBST in case of phosphorylated targets) and then incubated in PBS or TBS containing 0.02%(w/v) SDS for 1 h with Li-Cor secondary infrared antibodies at a 1:20000 dilution (Li-COR, Lincoln, NE). Membranes were washed three times for 5 min with PBST or TBST, and immunoreactive bands were visualized using a Li-COR Odyssey infrared imaging system (Li-COR, Lincoln, NE). Blots of crosslinked αSyn were visualized using ECL on an iBright system (Thermo Fisher Scientific, Waltham, MA).

The maturity of DIV21 iNs was also assessed using immunocytochemistry and confocal microscopy. Cells were fixed in 4% paraformaldehyde (PFA) and 4% sucrose at room temperature for 15 min and then permeabilized in PBS with 0.3% Triton X-100 for 10 min and blocked for 1 h in PBST with 5%(w/v) nonfat milk. iNs were incubated overnight with primary antibodies at 4°C, then washed 3 times with PBS and incubated for 1 h at room temperature with fluorescence-conjugated secondary antibodies (AlexaFluor 546 goat anti-mouse; and AlexaFluor 633 goat anti-rabbit). Finally, iNs were washed three times with PBS and examined using a Zeiss LSM710 confocal microscope fitted with a ×20 air objective (NA: 0.8). Maximal pixel intensity projections were created with averaging of 2 frames set to 1024 × 1024 pixel resolution. All antibodies used in this study, as well as the working dilutions are in Table 1.

**Table 1:** Antibodies and dilutions.

Antibody	Dilution	Catalog number
Alpha-synuclein	1:1000	MJFR1
Alpha-synuclein (phospho S129)	1:1000	EP1536Y
DJ-1	1:5000	AB18257
GAPDH	1:5000	EPR16891
SNAP25	1:5000	AB5666
PSD95	1:5000	MAB1598
Rab11	1:2000	AB65200
β3-tubulin	1:5000	AB18207
LRRK2	1:500	AB133474
Rab10	1:5000	8127 S
Rab10 (phospho T73)	1:100	AB241060
LRRK2 (immunofluorescence)	1:300	AB123474
Tyrosine hydroxylase (immunofluorescence)	1:600	AB150659

### DsiRNA-mediated knock-down

Predesigned Dicer-Substrate siRNA (DsiRNA) to target αSyn were ordered from IDT, and preparations were transfected into the neuronal cultures using Lipofectamine RNAiMAX Reagent (Thermo Fisher, 13778075) following the manufacturer’s protocol, the day after plating (day 5 post-induction).

### Statistical analysis

Data analysis was performed in the GraphPad Prism software v.9.0c. All data are expressed as the mean ± SEM. Data were analyzed for normal distribution using the D’Agostino-Pearson test. Statistical analysis was performed using one-way ANOVA with Holm-Sidak multiple testing correction (MTC) and Tukey’s post hoc test. Unpaired Student’s two-tailed *t* test or Mann–Whitney test was used when comparing between two groups. For each experiment, the test used is noted in the figure legend. A *p*-value of <0.05 was considered significant for all analyses. On each quantification blot, each dot (*N*) represents one experiment with 3–6 technical replicates (*n*).

## Supplementary information

Supplementary Information

## Data Availability

The authors declare that the main data supporting the findings of this study are available within the article and its Supplementary Information files. Extra data are available from the corresponding author upon request.
