# Supplementary material for: Parkinson-causing mutations in LRRK2 impair the physiological tetramerization of endogenous α-synuclein in human neurons
Source: NPJ Parkinsons Dis. 2022 Sep 16;8:118. doi: 10.1038/s41531-022-00380-1 (PMC9481630; doi:10.1038/s41531-022-00380-1)
Supplement: Supplementary file 1 — Supplementary Information [file 41531_2022_380_MOESM1_ESM.pdf]

Parkinson-causing mutations in LRRK2 impair the physiological tetramerization of endogenous α-synuclein in human neurons

Luis Fonseca-Ornelas, Jonathan M. S. Stricker, Stephanie Soriano-Cruz, Beatrice Weykopf, Ulf Dettmer, Christina Muratore, Clemens R. Scherzer and Dennis J. Selkoe*

Ann Romney Center for Neurologic Diseases

Department of Neurology

Brigham and Women's Hospital and

Harvard Medical School, Boston, MA 02115, USA

*Corresponding author: dselkoe@bwh.harvard.edu

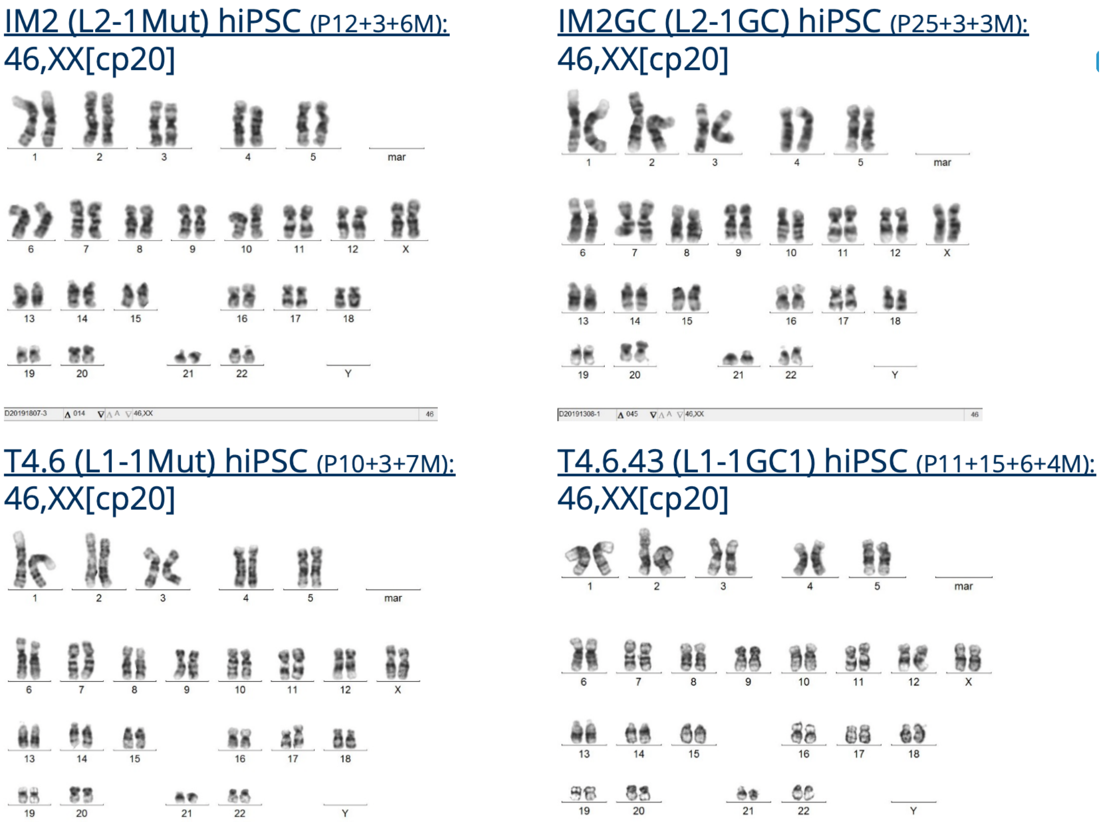

Supplemental Figure 1. LRRK2 mutant and corrected lines (L1 and L2) show normal karyotypes.

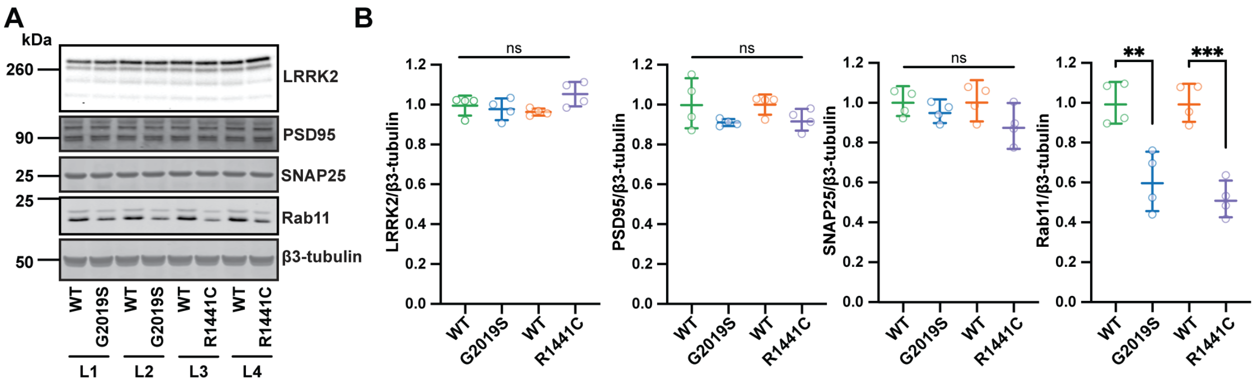

Supplemental Figure 2. Quantitative Western blotting of total protein extracts of LRRK2 WT and mutant neurons. (A) Representative WB of LRRK2 and neuronal maturation markers PSD95, SNAP25, β3-tubulin, and endocytic trafficking marker Rab11 with their (B) densitometric quantifications normalized to total levels of β3-tubulin in the same sample (N = 4; one-way ANOVA with Tukey's post hoc test; error bars = SD; **p<0.01, ***p<0.001, ns = not significant).

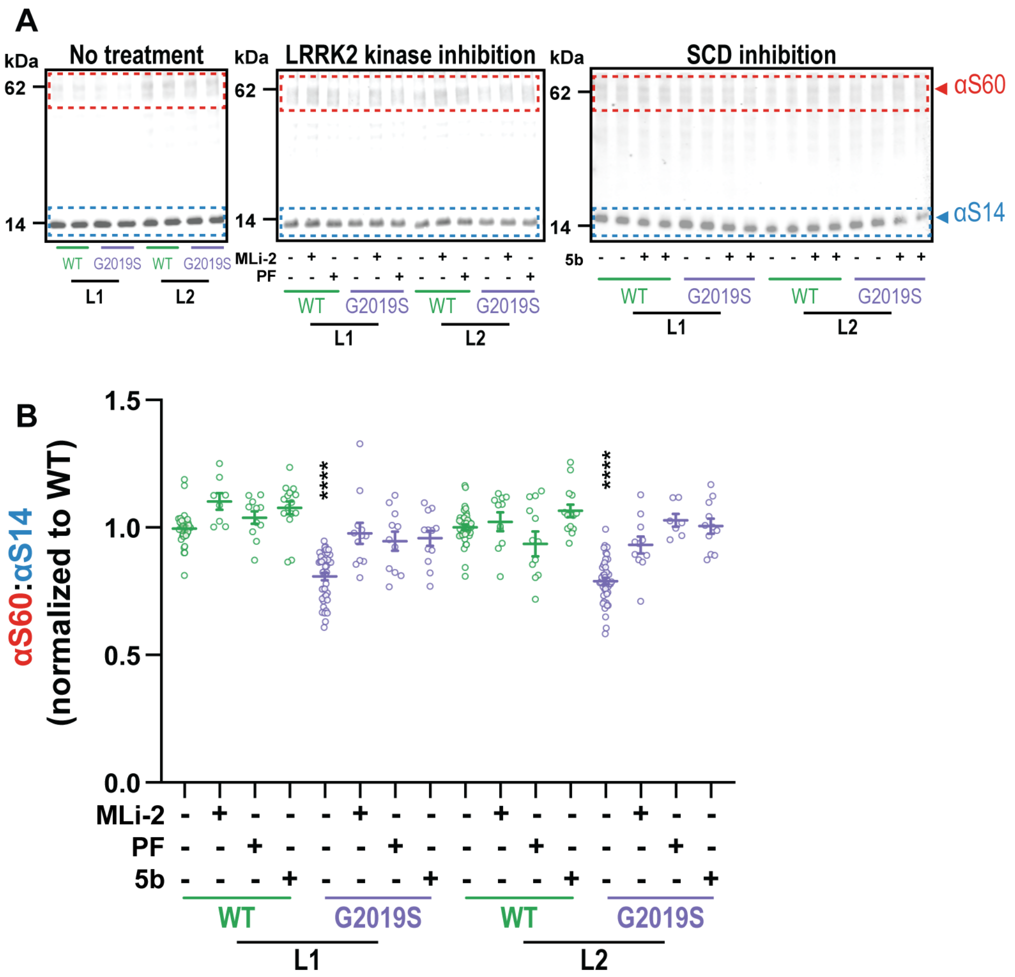

Supplemental Figure 3. Treatment with two LRRK2 kinase inhibitors (PF-06447475 (referred to as PF) and MLi-2) as well as an SCD inhibitor (5b) revert the lowered αSyn T:M ratio generated by the increased kinase activity of G2019S LRRK2. (A) Representative WBs of total cell lysates after intact-cell crosslinking after conditioning with (+) or without (-) PF compound (0.5 µM), MLi-2 (0.01 µM), and 5b (0.5 µM). (B) Quantification shows that compound conditioning increases the tetramer (αS 60) to monomer (αS 14) ratio of αSyn. Other than the untreated G2019S mutants, no other T:M ratio was significantly different to the untreated WT from both L1 and L2 isogenically corrected lines (N≥7 independent experiments; each dot is the quantification of the αSyn T:M ratio on a single gel lane –corresponding to a single crosslinked well; one-way ANOVA with Tukey's post hoc test; error bars = SEM; ****p<0.0001).

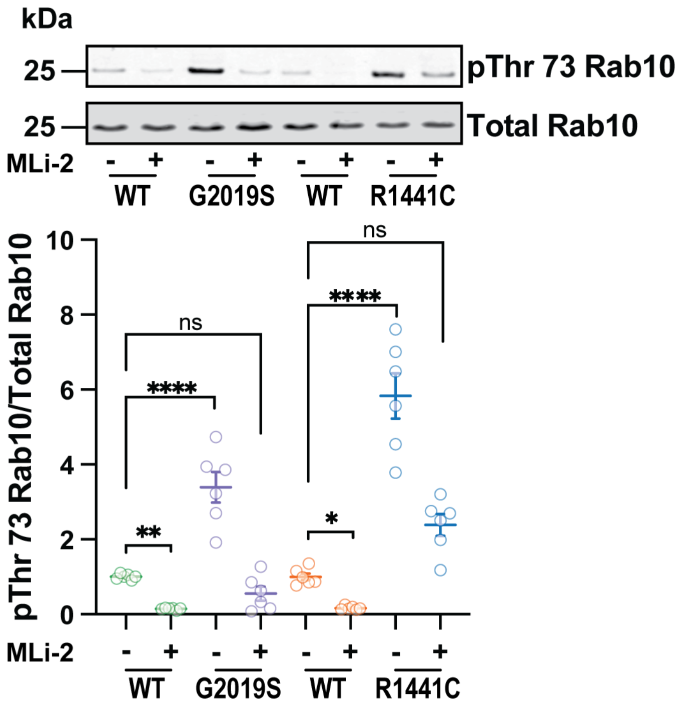

Supplemental Figure 4. The G2019S and R1441C mutations of endogenous LRRK2 in patient iNs enhance Rab10 phosphorylation. (A) Total cell lysates of iNs harboring the G2019S and R1441C mutations of LRRK2 compared to their isogenically-corrected controls (WT). (B) Both G2019S and R1441C mutations lead to hyperphosphorylation of Rab10 that is decreased with treatment by LRRK2 kinase inhibitor MLi-2 (**N** ≥6;one-way ANOVA with Tukey's post hoc test; error bars = SEM; *$p<0.05$, **$p<0.01$, ****$p<0.00001$, ns = not significant).

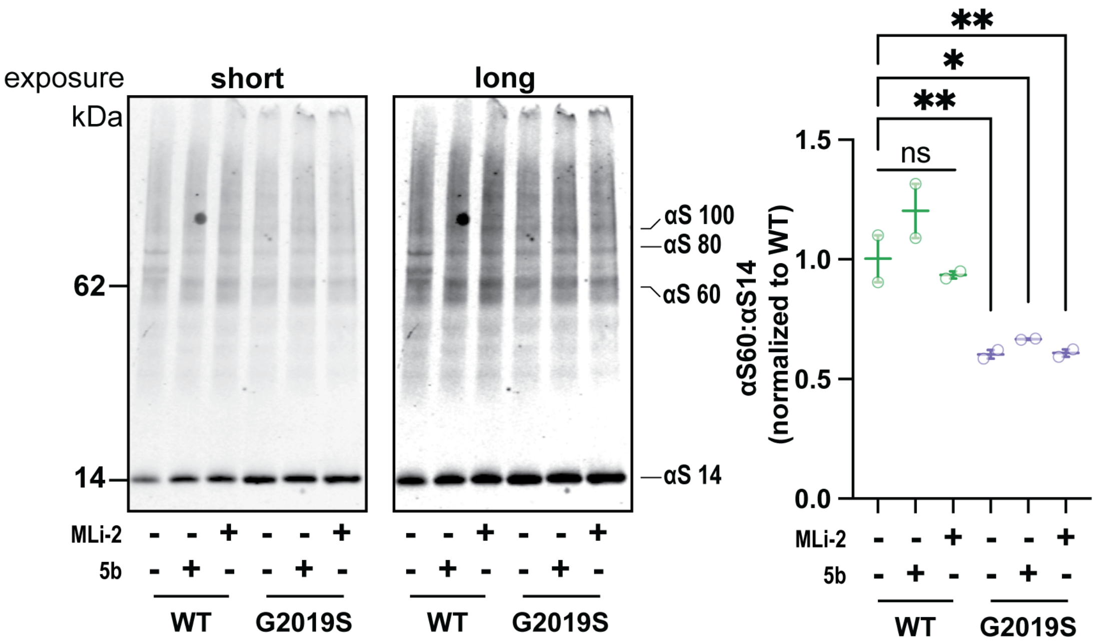

Supplemental Figure 5. Brief (~12 hr) RRK2 kinase inhibitor (MLi-2) treatment does not restore the αSyn T:M ratio. Left: short and long WB exposures of neuronal lysates after intact-cell crosslinking. Right: quantification of the blot (N=2). Neither MLi-2 nor the SCD inhibitor 5b restored the decreased T:M ratio of the G2019S neurons in 12 hr, in contrast to full restoration after 8 day treatment (see Figures 2-5, 7 and S3).

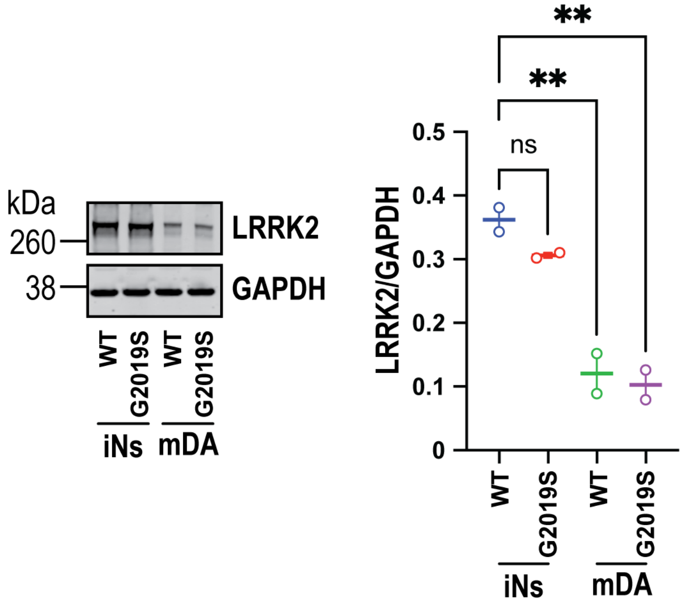

Supplemental Figure 6. WB of cell lysates from iNs and mDAs showing the expression levels of LRRK2 in the different cell types. While lower than in iNs, mDA neurons express LRRK2 in both its WT and mutant forms.

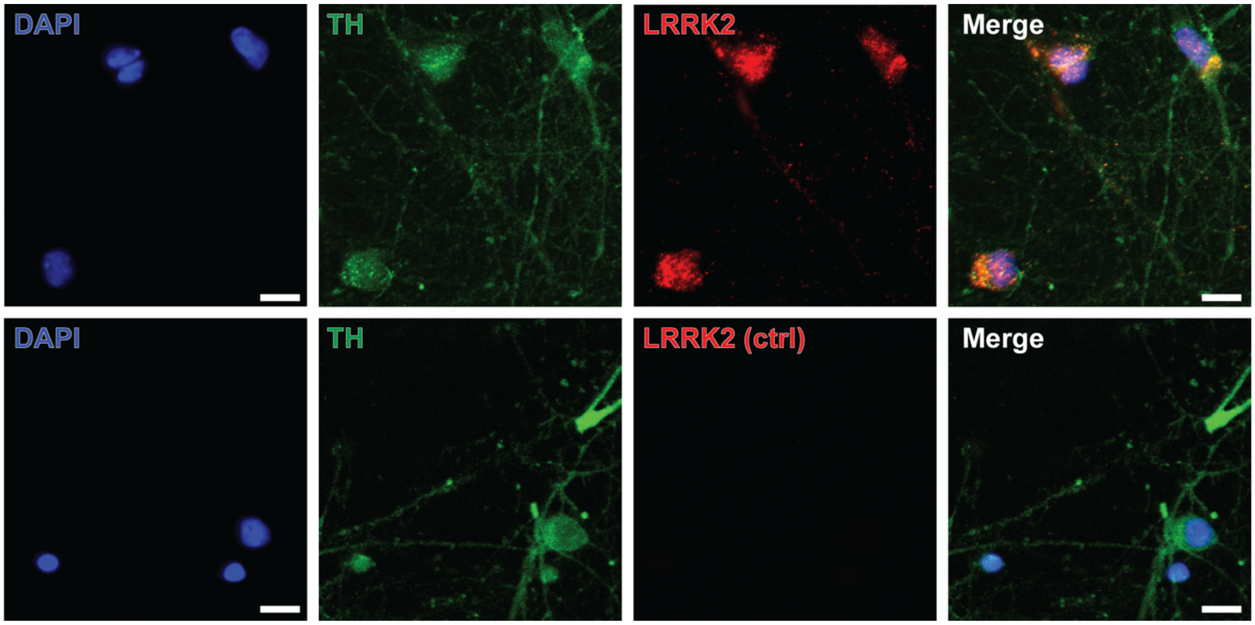

Supplemental Figure 7. Immunofluorescence reveals that dopaminergic neurons (TH positive, green) express LRRK2 (red). The bottom row shows a staining in the absence of LRRK2 primary antibody, demonstrating the reliability of the LRRK2 signal. The scale bar is 10 µm.
